# Fresh Osteochondral Allograft Transplantation in Osteochondritis Dissecans in the Knee Joint

**DOI:** 10.3390/life11111205

**Published:** 2021-11-08

**Authors:** Tommaso Roberti Di Sarsisa, Michele Fiore, Vito Coco, Marco Govoni, Leonardo Vivarelli, Nicola Rani, Nicolandrea Del Piccolo, Dante Dallari

**Affiliations:** Reconstructive Orthopaedic Surgery and Innovative Techniques—Musculoskeletal Tissue Bank, IRCCS Istituto Ortopedico Rizzoli, Via G.C. Pupilli 1, 40136 Bologna, Italy; tommaso.robertidisarsina@ior.it (T.R.D.S.); vito.coco@ior.it (V.C.); marco.govoni@ior.it (M.G.); leonardo.vivarelli@ior.it (L.V.); nicola.rani@ior.it (N.R.); nicolandrea.delpiccolo@ior.it (N.D.P.); dante.dallari@ior.it (D.D.)

**Keywords:** osteochondral allograft transplantation, osteochondritis dissecans, knee, lesion, healing

## Abstract

Osteochondritis dissecans (OCD) is a chronic and painful joint condition that can occur from childhood through to adult life. Microtrauma, vascular insufficiency, or abnormal endochondral ossification are the most common causes of OCD. Reconstructive techniques for OCD of the knee are typically necessary when either non-operative or reparative/regenerative operative treatments fail, or when the OCD is irreversible. To analyze the clinical outcomes and failure rates of fresh osteochondral allograft transplantation (FOCA) used as a reconstructive strategy in OCD patients, an in-depth search was carried out on the PubMed, Scopus, and Web of Science databases concerning the existing evidence related to the use of FOCA for OCD patients in the knee joint. A total of 646 studies were found through the search and 2 studies were added after a cross-referenced examination of the articles within the bibliography. Six studies with a total of 303 OCD lesions treated with FOCA, with a mean follow-up of 6.3 years, were included. Although a limited number of low-level evidence studies on this topic are available in previous research, satisfactory clinical results and survival rates of the reconstruction are reported. However, to better define the real advantages of FOCA in the healing process of OCD lesions, comparative studies with different techniques are needed.

## 1. Introduction

Osteochondritis dissecans (OCD) is an incompletely understood joint disorder affecting a broad spectrum of patients, but is most prevalent in adolescents and young adults [1]. The incidence of OCD is estimated to be approximately 15 to 30 per 100,000 patient-years [2,3]. OCD lesions are most frequently seen in the knee, occurring most often in the medial femoral condyle (70%, especially in the lateral aspect of the medial femoral condyle), followed by the lateral femoral condyle (15–20%), patella (5–10%), and trochlea (<1%) [4,5]. Although OCD was first described over 100 years ago, there is no consensus on its etiology. The original nomenclature suggested a major role for inflammation in OCD; however, histological evidence has failed to support this theory [6]. Current hypotheses on the origin of OCD include repetitive microtrauma, vascular insufficiency, or anomalous endochondral ossification [7]. This pathologic process involves the fragmentation of subchondral bone, which becomes avascular and detaches from the surrounding cartilage, often forming an intra-articular loose body [8]. The lesion can manifest as pain or through other symptoms, including catching and locking [2]. Age and skeletal maturity are important variables influencing clinical decision making because older, skeletally mature individuals (in the setting of lower healing potential) are less likely to succeed with non-operative treatment and more likely to progress to surgery [9,10].

The non-operative treatment of OCD with activity modification and bracing has been reported to be successful in 50% to 94% of patients with open physes and stable lesions [11,12]; therefore, most authors suggest initial non-operative treatment for juvenile OCD [9,13,14,15,16,17,18,19]. In the cases in which the physes are closed or the lesion is advanced—particularly in stage 3 (unstable but not dislocated fragment) or stage 4 (presence of loose body) according to the classification system proposed by Clanton and DeLee [20]—the success of non-operative treatment decreases [21]. Both reparative techniques, such as internal fixation [16,17,22], bone grafting [23], or debridement/fragment excision [24,25], and restoration techniques, such as anterograde/retrograde drilling [16,26,27] and autologous chondrocyte implantation (ACI) [28], have demonstrated variable healing outcomes. Large OCD de novo lesions, or those that progress after unsuccessful initial treatment and present with significant bone and cartilage defects, lead to long-term disability and are precursors to osteoarthritis at a young age [13,14]. These defects in children or adults should be considered for reconstructive treatment options, including various techniques using the bone of synthetic grafts associated with ACI [29,30], autologous osteochondral transplantation (OAT) or mosaicplasty (when multiple plugs are harvested to fill the defect in a mosaic-like pattern) [31,32,33,34] and osteochondral allograft (OCA).

Hypothetically, OCA is an attractive option because it can restore in a single-stage procedure both the bone and chondral components, potentially with neither the pitfalls of mosaicplasty (principally the morbidity of the donor zone of the knee, which limits the dimensions of the reconstruction), nor the high costs of the ACI-related procedures. The major indications for OCA transplantation include substantive joint surface compromise (>2 cm^2^) with bone loss and/or failed prior cartilage repair. Pathologic OCD tissue can be removed by cylindrical drills and replaced by press-fit “dowel grafts” (if necessary, fixation can be augmented with bioabsorbable screws or chondral darts) or resected to create a flat surface for the application of “shell grafts” [35]. The latter usually requires fixation to maintain compression (typically achieved by bioabsorbable or cannulated screws), although mixed methods have been described [36]. OCA can be fresh, frozen, cryo-preserved or freeze-dried tissue bank products. Stored allografts have shown reduced antigenicity and risk of disease transmission, but the preservation process also affects the biomechanical competency of the transplant [37,38]. Since it is relatively accepted that cartilage viability positively correlates with the integration of the graft, and consequently with the clinical outcome, fresh osteochondral allografts (FOCA) are preferred. FOCA transplantation procedures offer the primary advantage of containing viable hyaline cartilage and structurally competent bone. The term “fresh” refers to a graft harvested within 24 h of the donors’ death, stored (usually at 4 °C into an appropriate culture) until microbiological and viral tests are performed and then transplanted into a recipient host, usually within 28 days to avoid viability decrease [39].

The aim of this article is to collect research data regarding clinical outcomes, healing processes, and the reconstitution of survival rates from studies describe OCD patients treated with FOCA, paying particular attention to any differences based on the age and skeletal maturity of the patients, the size of the lesions, the type of FOCA reconstruction, and previous surgeries.

## 2. Materials and Methods

**Figure 1:**
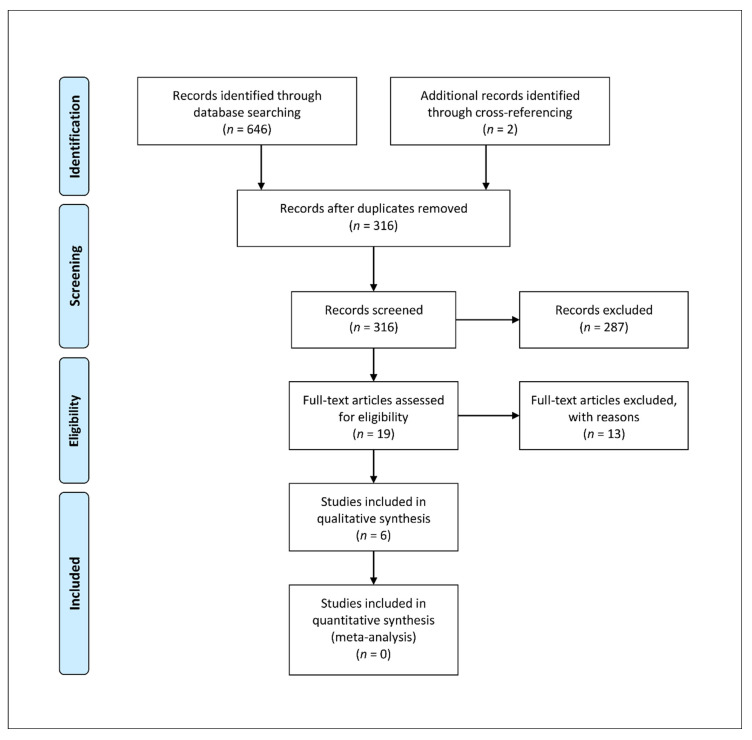
PRISMA ScR algorithm of the included studies.

An in-depth search of the scientific research was performed according to PRISMA (Preferred Reporting Items for Systematic Reviews and Meta-Analyses) Extension for Scoping Reviews (PRISMA ScR) [40]. The search algorithm according to these guidelines is shown in Figure 1. A search regarding the existing evidence for clinical healing outcomes and failure rates of FOCA transplantation of the knee joint in OCD population with no restriction on date of publication, up to the end of September 2021, was performed on the PubMed (https://pubmed.ncbi.nlm.nih.gov/ (accessed on 30 September 2021)), Scopus (https://www.scopus.com (accessed on 30 September 2021)), and Web of Science (www.webofscience.com (accessed on 30 September 2021)) databases. Various combinations of the following keywords were used: “osteochondral Allograft”, “osteochondritis dissecans”, “knee”. The inclusion criteria were as follows: original research reporting clinical outcomes and failure rates of FOCA for the treatment of osteochondritis dissecans in the knee joint, English language, minimum of five patients, minimum follow-up of 12 months, and human studies. The studies were categorized by study type, according to the Oxford Centre for Evidence-Based Medicine. We excluded animal studies, cadaveric studies, biomechanical reports, case reports, literature reviews, editorial articles, surgical technique descriptions, and instructional courses. Articles that were considered relevant during the electronic search were retrieved in full-text, and a cross-referencing hand-search of their bibliography was performed, in order to find further related articles. Reviews and meta-analysis were also analyzed, in order to broaden the search for studies that might have been missed through the electronic search. Only studies reporting data on homogeneous populations of patients with OCD, or from which data regarding patients with OCD were extractable, were included.

To assess the quality of the articles, the Institute of Health Economics (IHE) Quality Appraisal Checklist for Case Series Studies, which assesses methodologies based on 20 criteria (Table 1), was performed. Each study was assessed by two reviewers (M.F. and V.C.) independently and in duplicate; disagreement was resolved by the senior Author (D.D.).

The following data were independently extracted by all the investigators: demographics, diagnosis, length of follow-up, FOCA characteristics, concurrent treatment strategy, prior surgery, failure rate, reoperation rate, overall FOCA estimated survival rate (summarized in Table 2), and clinical scores (summarized in Table 3). Several clinical scores were reported in different studies to evaluate functional results. In this review, we considered the most recurrent scores: the 18 point, modified Merle d’Aubigné-Postel scale, the International Knee Documentation Committee (IKDC), the Knee Society Score (KSS), the Knee Injury and Osteoarthritis Outcome Score (KOOS), the Western Ontario and McMaster Universities Osteoarthritis (WOMAC) Index, the modified Hospital for Special Surgery (HSS) score, and the Visual Activity Score (VAS).

## 3. Results

A total of 646 studies were found through the electronic search and 2 studies were added after a cross-referenced research on the bibliography of the examined full-text articles. After a preliminary analysis, a total of six studies were included in this scoping review [2,41,42,43,44,45] (Figure 1). Of the included studies, all had Level of Evidence IV; five were retrospective case series [2,41,42,44,45] and one was a prospective case series [43]. The overall quality of the case series assessed via the IHE checklist, resulted as high in four cases [41,42,43,44], moderate [2] and low [45] in the remaining two studies (Table 1).

### 3.1. Patients’ Characteristics

As shown in Table 2, a total of 280 patients and 303 OCD lesions treated with FOCA was included. In the studies analyzed, the medium follow-up ranged from 2 years (range, 1–3.4) [42] to 7.7 years (range, 2–22) [44], with an approximated weighted mean of 6.3 years. The medium age at surgery ranged from 15.2 years (range, 13–20.4) [42] to 34 years (range, 20–49) [43], with an approximated weighted mean of 23.9 years. The location of the OCD lesions reported in the analyzed studies was predominantly at the level of the medial and lateral condyles of the distal femur, in a similar ratio. Lyon et al. and Sadr et al. also included a substantial proportion of OCD lesions at the level of the patella (7.5% and 1%, respectively) and trochlea (7.5% and 6%, respectively) [2,42]. The mean size of the OCD defects (reported in 5 studies [2,41,42,43,44]) was high, ranging from 4.5 cm^2^ [43] (range, 0.9–15) to 7 cm^2^ [44], with an approximated weighted mean of 6.7 cm^2^. Only the study of Lyon et al. [42], on 11 patients, focused on juvenile OCD; however, no studies specified the exact number of patients with open physes, who were intended to be a very restricted minority of the total number of patients included in this review. Four studies (contributing to the large majority of the patients included in this review) [2,41,44,45] described series in which all or most of the included patients had undergone previous surgery (included previous grafts), before FOCA transplantation (Table S1). Concomitant surgeries were described in three studies [41,43,44] (Table S1).

### 3.2. Graft Characteristics

Four studies specified how the graft was stored (Table S1), in all cases at 4 °C in appropriate culture fluid [2,41,44,45]. The time between graft harvesting and transplantation was reported in four studies and ranged from 5 to 28 days [2,41,42,44] (Table S1). Graft size was rarely specified; however, authors reported the use of dowels rather than shells in most cases (Table S1). Dowels were typically press-fit, while absorbable fixation was more often used for shells.

### 3.3. Graft Survival

The definition of reconstruction failure varied across studies (Table S1). In general, studies with shorter follow-up used clinical failure or radiological non-integration of the graft as criteria. By contrast, studies with a longer follow-up defined failure as the revision of the reconstruction or conversion to unicompartmental or total knee arthroplasty. The failure rate at last follow-up ranged from 0% (reported by Lyon et al. [42] with a mean follow-up of 2 years) to 13% (reported by Emmerson et al. [44] with a mean follow-up of 7.7 years). Cotter et al. [41] reported a 97% estimated reconstruction survival rate (RSR) at 5 years on 43 FOCA transplantations; Sadr et al. [2] reported 95% RSR at 5 years and 93% at 10 years on 149 FOCA; Emmerson et al. [44] reported 91% RSR at 5 years and 76% RSR at 10 and 15 years on 66 FOCA; while Lyon et al. [42], Pascual-Garrido et al. [43] and Garrett et al. [45] reported graft survival rates at last follow-up of 100%, 94%, and 94%, respectively. In the studies of Sadr et al. [2] and Emmerson et al. [44], the age at surgery were reported to be higher and the OCD lesion size was larger in the subgroups who received revision surgery due to graft failure. In the study by Sadr et al. [2], the median age and the mean lesion size were 31 years and 7.6 ± 2.8 cm^2^ in the revised patients versus 21 years and 7.3 ± 3.3 cm^2^ in the total cohort, while in the study of Emmerson et al. [44], the mean age and the mean lesion size were 32.9 ± 10.6 years and 11.3 ± 4.7 cm^2^ in the revised patients versus 28.6 years and 7.5 cm^2^ in the total cohort. The mean time to failure was reported in five studies [2,41,43]: Cotter et al. [41] reported a mean time to failure of 6.2 ± 3.8 years (mean follow-up 7.29 ± 3.3 years), Sadr et al. [2] reported 6.1 ± 4.5 years (mean follow-up 6.3 years, ranging from 1.9 to 16.8), Emmerson et al. [44] reported 4.9 ± 2.4 years (mean follow-up 7.7 years, ranging from 2 to 22), while both Pascual-Garrido et al. [43] and Garrett et al. [45] reported a single failure at 14 months and 15 months after surgery, respectively.

### 3.4. Functional Outcomes

As reported in Table 3, five studies reported the results of at least two clinical scores administered to patients pre- and post-operatively [2,41,42,43,44]. In all cases, better scores were observed after surgery, with the majority of differences being statistically significant. Four studies reported the percentage of patients who were satisfied overall, which ranged from 63% to 95% [2,41,43,44]. A more comprehensive overview of the results of the most frequently used clinical scores in the analyzed studies is provided in Table 3 and Table S2.

## 4. Discussion

This review aims to gather evidence on the results of FOCA in the treatment of OCD, to understand whether this pathology may represent a niche of particular interest in the use of FOCA. However, a brief overview of the treatment scenario of OCD and other osteochondral lesions of the knee is necessary.

The management of deep and large OCDs to avoid arthroplasty in young patients represents a challenge. Cartilage restoration techniques, such as microfracture and autologous chondrocyte implantation (ACI), do not replace bone defects and are less suitable procedures when the underlying bone is damaged. Hence, apart from OCA, other treatment options principally include various combinations of morselized bone graft and synthetic grafts covered with an ACI patch, and osteochondral autograft transplantation (OAT)/mosaicplasty [46].

ACI and ACI-related procedures are two-stage cell-based autograft techniques [46,47]. The first stage involves an arthroscopic biopsy from healthy cartilage in the non-weight-bearing region of the intercondylar notch. The harvested cells are grown in vitro over 4–6 weeks, when the patient returns for implantation. Many authors have reviewed ACI plus bone grafting and have reported good or excellent results in 73–86% of patients [47,48,49]. Specifically, Carey et al., evaluating 67 juvenile OCD lesions with a mean size of 6 cm^2^ treated with ACI ± bone grafts, found an estimated failure rate of 87% at 10 years and 82% at 20 years, defining the failure as the revision of the graft or conversion to arthroplasty [30]. Third-generation techniques, termed MACI (matrix-induced autologous chondrocyte implantation), impregnate the chondrocytes into the collagen matrix in vitro, rather than the matrix being applied on top of cellular material. Roffi et al., in a recent prospective case series evaluating 19 patients who underwent MACI in OCD lesion with a mean size of 2.8 cm^2^, found a failure rate of 16% at 10 years of follow-up, with worse subjective results in patients with lesions >3.5 cm^2^ [29]. The current NICE (National Institute of Health and Care Excellence) guidelines recommend MACI as an option for treating symptomatic grade 3 or 4 defects >2 cm^2^ in patients who have minimal arthritic changes and no previous surgical repairs to the articular cartilage [50]. Moreover, it should be emphasized that regenerative techniques using autologous chondrocytes or mesenchymal stem cells show the greatest potential for future development [51]. The optimization of these techniques by (1) the acquisition of new knowledge regarding the specific stem cell populations with the greatest chondrogenic potential, (2) the introduction of new scaffolds and biomaterials capable of providing a more stable construct and a personalized osteochondral defect reconstruction, and (3) the implementation of the use of new biological stimuli, may improve their results and extend their surgical indications to larger osteochondral lesions. However, currently, all these options are thought to be more expensive than OCA and only a few studies have evaluated results on very large osteochondral defects [46].

Mosaicplasty involves the removal of osteochondral cylinders from a region with low load impact (usually the lateral margin of the femoral trochlea or the area above the intercondylar sulcus) and transferring them to the area of the lesion. The advantages of OAT include a single-stage procedure that is usually performed arthroscopically, the use of hyaline cartilage with its superior mechanical properties to fibrocartilage, and the ability to address both subchondral bone loss and articular cartilage defects. The disadvantages of the procedure include donor-site morbidity, size mismatching, and a limited area available for harvesting [52]. The use of OAT for the treatment of OCD lesions was first described in 1985 [34]. Since then, encouraging results have been reported [53,54,55,56]. However, the results have appeared to be highly dependent on lesion size [57]. In fact, while Smolders et al. [54] described satisfactory results treating OCD lesions ranging from 0.5 to 3.2 cm^2^, and other studies reported that OAT provides good-to-excellent results when applied to smaller articular cartilage between 1 and 4 cm^2^ [58], lesions > 6 cm^2^ are associated with a poor prognosis, even when multiple graft plugs are used [53]. Furthermore, few studies with small cohorts have looked specifically at the use of OAT for the treatment of OCD [32,54,59,60,61].

A recent meta-analysis by Zamborsky et al. [62] aimed to compile data on all the results of RCTs on microfracture, OAT, ACI and MACI in knee osteochondral lesions, with a mean size ranging between 2.1 cm^2^ and 6.1 cm^2^. Using data from 21 RCTs, they found that the re-operation, failure, and adverse event rates were similar for all procedures. However, microfractures demonstrated the worst patient-reported outcomes and poor long-term results. They concluded that cartilage repair techniques provided higher quality repair of tissue, lower failure rates, and higher return-to-activity rates, recommending ACI as the best intervention, followed by OAT [62]. Unfortunately, no RCTs have been conducted on OCA and, hence, outcomes of this treatment were not included for comparison. Research on the topic is largely composed of historical case series, with a distinct lack of comparative studies or available modern papers suggesting that the development of restorative techniques has reduced the demand for this procedure. Therefore, the lack of high-level evidence does not allow the recommendation of OCA as the treatment of choice in every scenario. The trend in the relevant research seems to be to reserve OCA primarily (though not exclusively) for the treatment of very large osteochondral lesions or revision surgery. Therefore, FOCA, with the advantage of its ability to create an exact size match for the lesion without compromising the donor site, in the setting of a single-stage procedure, remains a versatile treatment. It is recommended for medium and deep, large, or very large unsalvageable osteochondral lesions or revision procedures, thus representing a viable option in the setting in which most other treatments fail or are not fully recommended due to lack of evidence.

The possible disadvantages of FOCA include its cost and graft availability, as well as the potential for disease transmission and graft-host immunological reactions [63].

Regarding the costs to the healthcare system, a recent review by Mistry et al. shows that OCA transplantation in the management of osteochondral lesions appears to be highly cost-effective in preventing increased healthcare costs related to other potential treatments or their failure [46].

On the other hand, a major limitation of this technology is the availability of tissue. Regulatory restrictions, organization, and distribution issues combine to limit the possible application and benefits of this successful procedure to a few countries [36]. Moreover, the short period of time between the identification of an appropriate tissue and surgery, with the resulting restrictions on the patient’s daily activities due to postoperative rehabilitation, also limit surgical scheduling and patient acceptance [64].

Extensive serological, bacterial, and viral testing of grafts is necessary prior to allograft transplantation and donors must be screened [65,66,67]. However, with the implementation of nucleic acids analysis, the risks linked to the window of infectivity have decreased for all the most dangerous viruses, such as HCV and HIV (the risk of HIV transmission is estimated to be as low as approximately 1 in 1.6 million, and there have been no reports of this route of disease transmission since the late 1980s),but not for all viruses or donors affected by emerging diseases [65,66].

Although there is no specific concern over systemic immunologic “rejection” phenomenon, and blood type matching is not required for this process, limiting the potential for antigenic exposure with abundant washing and removal of residual donor soft tissue is strongly recommended to minimize immunogenic-guided resorption [37,68,69]. In fact, while chondrocytes are preserved against immunological reactions by the matrix cells, the cells in the bony part of the graft are exposed to the host reaction, representing a potential cause of failure [65]. Hence, Hunt et al. [70] retrospectively analyzed whether the development of antibodies against HLA was related to the size of the knee FOCA. They found HLA positivity in 70%, 54%, and 6% of patients receiving large, medium, and small FOCA, respectively. The difference was statistically significant between the large (>10 cm^2^) and the small (nearly 6.5 cm^2^) groups. Despite this, between HLA-positive and -negative groups there were no significant differences in failure rate, time to failure, graft area and type or location, suggesting that HLA positivity does not correlate with clinical outcome [70].

Despite some of the possible limitations discussed above, the advantages of this procedure remain numerous and encouraging results have been reported in research. The review by Familiari et al. [71] identified 19 studies with a total of 1036 patients in which OCA transplantation was used in primary and revision procedures to treat different types of knee osteochondral defect, with a weighted mean follow-up of 8.7 years (range, 2–32 years). The mean 5-year survival rate across the studies included in their review was 86.7%, while the mean 10-year and 20-year survival rate was 78.7% and 67.5%, respectively. The weighted mean reoperation rate was 30.2% (range, 0–63%) and the mean failure rate was 18.2% (range, 0–31%). However, the size of the lesions was reported in a minority of the studies included. The authors further note that revision cases, patellar lesions, and bipolar lesions demonstrated worse survival rates [71]. Tschon et al. [39], in a more recent review, retrieving 18 papers on the use of FOCA in knee surgery, found a total of 769 FOCA implanted in 744 patients for the treatment of deep and large or very large osteochondral defects (the mean size ranged between 4.8 cm^2^ and 19.2 cm^2^), including traumatic or degenerative osteochondral lesions, such as those caused by post-traumatic or idiopathic osteoarthritis, osteonecrosis, osteochondritis dissecans, avascular necrosis, or previously failed treatments. The patients were followed-up for an average of 7 years (range 1.6–13.5 years). After excluding a study investigating the use of FOCA in end-stage knee post-traumatic arthritis [72], the re-operation rates ranged from 0% to 34%, while the reported failure rates ranged from 0% to 45.8%, although the definition of failure differed among the authors. In particular, two studies included in the review by Tschon et al. [39] reported high rates of return-to-sport (RTS) after the treatment of focal cartilage lesions of the knee of any etiology with FOCA [73,74]. Krych et al. [73] noted an 88% RTS rate at an average of 9.6 months, with 79% achieving the preinjury activity level, in a study of 43 patients. Nielsen et al. [74] conducted a larger RTS study after OCA with 149 knees included: a total of 112 (75.2%) were able to RTS or return to recreational activity after FOCA, with 91% of athletes reporting being satisfied or extremely satisfied with their clinical outcome. On this topic, a recent review by Crawford et al. [75] of 13 studies suggests that FOCA transplantation for cartilage defects allows most athletes to return to sport (range, 75%–82%). Most of the studies included reported improvements in sports-specific patient-reported outcomes at follow-up and reached the minimal clinically significant difference. However, the re-operation rate was high in several studies (ranging from 34% to 53% in more than half of the studies), with a large percentage of patients requiring loose body removal or debridement. The long-term survival of the allografts was found to be largely unknown; however, the authors concluded that FOCA transplantation consistently improves function in athletes with chondral injuries [75]. Another systematic review, by Chahla et al. [76], focused on the use of various types of FOCA for osteochondral lesions of the patella-femoral joint. They identified 8 studies with a total of 129 patients, finding a mean survival rate of 87.9% at 5 years and 77.2% at 10 years [76].

On the risk factors for FOCA failure, Frank et al. [77], on 224 consecutive patients undergoing FOCA for any indication, reported a greater BMI as the only independent predictor of failure, while Levy et al. [78], evaluating 129 knees, found a significant association between age over 30 years at the time of surgery and allograft failure. In fact, regarding age as a prognostic factor, several studies in pediatric and adolescent patients have reported better results than studies with adult populations [42,79,80]. Murphy et al. [79] reported on a case series of 38 patients (43 knees) younger than 18 years at the time of surgery undergoing FOCA transplantation, with a mean follow-up period of 8.4 years (range, 1.7–27.1 years). They found 89% of patients to be satisfied or extremely satisfied with the procedure, with a graft survivorship rate of 90% at 10 years. Lyon et al. [42] retrospectively reviewed a case series of 11 patients (mean age, 15.2 years) with OCD undergoing FOCA transplantation. They reported a 100% graft survival with a mean follow-up of 24 months. More recently, Gilat et al. [81] evaluated 36 patients younger than 18 years, with a mean follow-up of 4.6 years. They found a failure rate of 5.6% and a re-operation rate of 28.8%. Moreover, Horton et al. [82] recently reported on the long-term outcome of revision allografting, with a 61% survivorship at 10 years, suggesting that revising a failed allograft is an appropriate intervention.

Definitively, with the limitations posed by the scarce high-quality evidence and the lack of comparative studies, the available research suggests that the medium- and long-term results of FOCA in the treatment of osteochondral lesions of the knee would seem to be slightly inferior to those obtained by restorative techniques; however, the apparently different percentage of very large lesions included should be considered.

Focusing on advanced OCD, the results of FOCA are among the most favorable seen among the surgical treatments, although direct comparison is confounded by patient age, lesion size, lesion location and associated diagnosis (malalignment, knee instability, meniscal pathology) [78].

Overall, the studies included in this review showed excellent clinical outcomes, although these are poorly comparable because the outcome scores used differ substantially between studies.

The survival of reconstruction has been described as 91% to 100% in short-term follow-up studies (up to five years). Only the two studies with the largest number of patients, by Sadr et al. [2] and Emmerson et al. [44], evaluated estimated survival rates beyond ten years (both using graft revision or conversion to arthroplasty as definition of failure), and reported 93% and 76% survival, respectively. This difference could be partially explained by the higher mean of surgical treatments prior to FOCA and the higher age of the patients in the Emmerson et al. case series. Interestingly, in both the studies by Sadr et al. [2] and Emmerson et al. [44] the median and mean age of patients, respectively, who underwent revision surgery after graft failure, were higher than the mean/median age of the total cohort (31 vs. 21 years and 32.9 vs. 28.6 years, respectively). However, these remain the only possible speculations regarding the different outcomes according to different patient ages, as the two studies with the lowest and highest mean age (Lyon et al. [42] and Pascual-Garrido et al. [43], respectively), reported data on a very low number of patients and too short a follow-up to detect any potential differences. Similarly, with regard to OCD lesion size, only the studies by Sadr et al. [2] and Emmerson et al. [44] showed potential differences in outcome. In fact, in both these studies, the patients who underwent revision surgery after graft failure had larger lesions before FOCA than the mean of the total cohort (7.6 vs. 7.3 cm^2^ and 11.3 vs. 7.5 cm^2^, respectively). However, it should be noted that the overall mean size of the OCD lesions treated in the studies included in this review is considerably high (6.7 cm^2^). As mentioned above, lesion size is a crucial factor in the choice of treatment for all unsalvageable osteochondral lesions, including OCD lesions. This is particularly important when attempting to compare with research data on the results of other types of treatment in the management of large osteochondral lesions. In this regard, another element worth considering is the high percentage of patients in the studies included in this review who underwent FOCA as a second or third procedure following previous failed treatments.

The findings of this review are highly limited by the paucity and low quality of the included studies; however we can conclude that, according to the good clinical results and survival rates of FOCA transplantation described in the studies included, despite the high average size of the treated OCD lesions, future high-quality comparative studies between FOCA and other osteochondral defect reconstruction techniques are desirable in order to define the possible advantages of the use of FOCA in some categories of patients (e.g., very large OCD lesions or multi-treated patients).

## 5. Conclusions

Fresh osteochondral allograft transplantation for irreversible osteochondritis dissecans lesions of the knee resulted, among the majority of patients, in significant improvements in pain and function with surviving grafts in the studies analyzed. Allografts also demonstrated good long-term durability, with high survivorship. The failure of previous treatments or allografts did not preclude revision allografting. Despite the very significant limitations imposed by the paucity and low quality of the available evidence, it can be concluded that this technique appears to be a safe and effective in the treatment of medium and large osteochondritis dissecans, representing a valid option to promote healing. Nevertheless, age at surgery and the size of the OCD lesion could affect graft survival, although there is insufficient data to state this definitively. The available research seems to suggest that the choice of FOCA can also be guided by the size of the lesion in the setting of OCD. However, only high-quality comparative studies with other techniques could define the possible and real advantages of FOCA.

## Figures and Tables

**Table 1:** IHE quality appraisal checklist for case series included in this review.

Study	Cotter et al., 2018	Sadr et al., 2016	Lyon et al., 2012	Pasqual-Garrido et al., 2009	Emmerson et al., 2007	Garrett et al., 1994
Q1: was the hypothesis/aim/objective of the study clearly stated?	yes	yes	yes	yes	yes	yes
Q2: was the study conducted prospectively?	no	no	no	yes	no	no
Q3: were the cases collected in more than one centre?	no	no	yes	no	no	no
Q4: were patients recruited consecutively?	unclear	unclear	unclear	yes	unclear	yes
Q5: were the characteristics of the patients included in the study described?	yes	yes	yes	yes	yes	yes
Q6: were the eligibility criteria (i.e., inclusion and exclusion criteria) for entry into the study clearly stated?	yes	no	yes	no	no	no
Q7: did patients enter the study at a similar point in the disease?	yes	yes	yes	yes	yes	yes
Q8: was the intervention of interest clearly described?	yes	yes	yes	no	yes	yes
Q9: were additional interventions (co-interventions) clearly described?	yes	yes	yes	yes	yes	yes
Q10: were relevant outcome measures established a priori?	yes	yes	yes	yes	yes	no
Q11: were outcome assessors blinded to the intervention that patients received?	unclear	unclear	no	unclear	unclear	unclear
Q12: were the relevant outcomes measured using appropriate objective/subjective methods?	yes	yes	yes	yes	yes	no
Q13: were the relevant outcome measures made before and after the intervention?	yes	yes	yes	yes	yes	no
Q14: were the statistical tests used to assess the relevant outcomes appropriate?	yes	no	no	yes	yes	no
Q15: was follow-up long enough for important events and outcomes to occur?	yes	yes	yes	yes	yes	yes
Q16: were losses to follow-up reported?	yes	no	yes	yes	yes	no
Q17: did the study provided estimates of random variability in the data analysis of relevant outcomes?	yes	yes	yes	no	yes	no
Q18: were the adverse events reported?	yes	yes	yes	yes	yes	yes
Q19: were the conclusions of the study supported by results?	yes	yes	yes	yes	yes	yes
Q20: were both competing interests and sources of support for the study reported?	yes	yes	yes	yes	yes	no
TOTAL (yes/no/unclear)	16/2/2	13/5/2	16/3/1	15/4/1	15/3/2	9/10/1

**Table 2:** Fresh osteochondral allograft transplantation in the treatment of osteochondritis dissecans.

Year	Authors [Reference]	Patient, n° (OCA, n°)	Study Design (Level of Evidence)	Knee Site (%)	Age, y: Mean ± SD (Range)	FU, y: Mean ± SD (Range)	Lesion Size, cm^2^: Mean ± SD (Range)	Failure at Last FU, n° (%)	Estimated Graft Survival Rate	Re-Operation Rate *	Mean Time to Failure, y Mean ± SD
2018	Cotter et al. [39]	37 (43)	Case series (IV)	LFC 44% MFC 51% Both condyles 4%	26 ± 9.96 (15–49)	7.29 ± 3.3	4.6 ± 1.7	2 (5.1%)	97% at 5 years	35.9%	6.2 ± 3.8
2016	Sadr et al. [2]	135 (149)	Case series (IV)	MFC 62% LFC 29% Trochlea 6% Patella 1% Others 2%	Median. 21 (12–55)	Median: 6.3 (1.9–16.8)	7.3 (2.2–25)	12 (8%): 7 OCA revision, 3 UKA, 2 TKA	95% at 5 years 93% at 10 years	23%	6.1 ± 4.5
2012	Lyon et al. [40]	11 (12)	Case series (IV)	MFC 31% LFC 54% Patella 7.5% Trochlea 7.5%	15.2 (13–20.4)	2 (1–3.4)	5.1 (1.8–8)	0%	100% at last FU	0%	NA
2009	Pasqual-Garrido et al. [41]	46 (16)	Case series (IV)	NA	34 ± 9.5 (20–49) **	4.0 ± 1.8 (2.0–10.6) **	4.5 ± 2.7 (0.9–15) **	1/16 OCA (6%): TKA	94% at last FU **	NA	14 months
2007	Emmerson et al. [42]	64 (66)	Case series (IV)	MFC 62% LFC 38%	28.6 (15–54)	7.7 (2–22)	7.5	9 (13%): 6 OCA revision, 1 OCA removal, 1TKA, 1 UKA	91% at 5 years 76% at 10 and 15 years	10 (15%)	4.9 ± 2.4
1994	Garrett et al. [43]	17 (17)	Case series (IV)	LFC 100%	20 (16–46)	3.5 (2–9)	NA	1 (6%): not specified reconstructive surgery	94% at last FU	17 (100%): 1 failure + 16 hardware removal	15 months

Abbreviations: OCD, osteochondritis dissecans; OCA, osteochondral allograft transplantation; MFC, medial femoral condyle; LFC, lateral femoral condyle; TKA, total knee arthroplasty; UKA, unilateral knee arthroplasty; FU, follow-up; NA, not available. * Re-operation rate = failures + operations not related to the graft; ** On total study cohort.

**Table 3:** Clinical scores reported in at least two of the studies included in this review.

		Cotter et al., 2018	Sadr et al., 2016	Lyon et al., 2012	Pasqual-Garrido et al., 2009	Emmerson et al., 2007
18-point		NA	Pr: 13.6 (±2.0) F: 16.8 (±1.5) *p*: <0.001 *	Pr: 12.7 (10–14) F: 16.3 (10–18)	NA	Pr: 13.0 ± 1.7 F: 16.4 ± 2.0 *p*: <0.01 *
IKDC total score		Pr: 31 F: 59 *p*: <0.001 *	Pr: 44.2 (± =17.5) F: 82.3 (± =15.8) *p*: <0.001 *	NA	Pr: 31 F: 45 *p*: 0.15	NA
KOOS	Symptoms	Pr: ≈52 F: ≈69 *p*: <0.001 *	NA	NA	Pr: 52 F: 74 *p*: 0.002 *	NA
	Pain	Pr: ≈50 F: ≈70 *p*: <0.001 *			Pr: 59 F: 67 *p*: 0.270	
	ADL	Pr: ≈61 F: ≈82 *p*: <0.001 *			Pr: 57 F: 67 *p*: 0.200	
	Sport	Pr: ≈23 F: ≈51 *p*: <0.001 *			Pr: 32 F: 46 *p*: 0.037 *	
	QOL	Pr: ≈21 F: ≈51 *p*: <0.001 *			Pr: 29 F: 39 *p*: 0.062	
SF-12	Physical	Pr: ≈33 F: ≈41 *p*: <0.001	NA	NA	Pr: 42 F: 52 *p*: 0.112	NA
	Mental	Pr: ≈53 F: ≈53 *p*: 0.910			Pr: 40 F: 43 *p*: 0.370	
VAS		NA	NA	Pr: 5.6 F: 1.2	NA	Pr: 6.7 ± 2 F: 0.9 ± 1.3
Satisfaction at Final FU, % (details)		81% (Es: 50%; S: 31.6%)	95% (Es: 78%; S: 17%; Ss: 3%; Sd: 1%; D: 1%)	NA	63%	92%

Abbreviations: Pr, preop. value; F, final FU value; *p*, *p*-value; 18 point, modified Merle d’Aubigné-Postel scale; IKDC, International Knee Documentation Committee; KOOS, knee injury and osteoarthritis outcome score; QOL, quality of life; ADL, activities of daily living; SF-12, 12 Item Short Form Survey; VAS, visual activity score; Es, extremely satisfied; S, satisfied; Ss, somewhat satisfied; Sd, somewhat dissatisfied; D, dissatisfied; NA, not available. * Statistically significant.

## Data Availability

No new data were created or analyzed in this study. Data sharing is not applicable to this article.

## Supplementary Materials

The following are available online at https://www.mdpi.com/article/10.3390/life11111205/s1. Table S1: Fresh osteochondral allograft transplantation in the treatment of osteochondritis dissecans (extended version). Table S2: Clinical scores reported in the included studies of this review.
