# Supplementary material for: Fresh Osteochondral Allograft Transplantation in Osteochondritis Dissecans in the Knee Joint
Source: Life (Basel). 2021 Nov 8;11(11):1205. doi: 10.3390/life11111205 (PMC8622509; doi:10.3390/life11111205)
Supplement: Supplementary file 1 [file life-11-01205-s001.zip › Table S1.pdf]

Table S1. Fresh osteochondral allograft transplantation in the treatment of osteochondritis dissecans (extended version).

Year	Authors [reference]	Patient, n° (OCA, n°)	Study design (Level of Evidence)	Knee site (%)	Age, y: mean ± SD (range)	FU, y: mean ± SD (range)	Lesion Size, cm²: mean ± SD (range)	FOCA storage time and conditions	FOCA type, n°; Mean size, cm²: mean ± SD (range)	FOCA Fixation technique, n°	Prior Surgery, n° (%) / Procedures per Patient, n° (Mean);	Concomitant procedures	Definition of Failure	Failure at last FU, n° (%)	Estimated graft survival rate	Re-operation rate*	Mean time to failure, y Mean ± SD
**2018**	**Cotter et al.** [39]	37 (43)	Case series (IV)	LFC 44%<br>MFC 51%<br>Both condyles 4%	26 ± 9.96 (15-49)	7.29 ± 3.3	4.6 ± 1.7	Grafts preserved at 4°C	Dowel, 41<br>Snowman, 2	Most common press-fit	OCD ORIF, 17 (43.5%)<br>LBR, 13 (32.5%)<br>Microfracture and/or drilling, 21 (52.5%)<br>ACI, 1 (2.5%)<br>OATS, 1 (2.5%)	11: 6 MAT, 3 HTO, 4 DFO	OCA Revision, gross appearance of graft failure on second-look arthroscopy, knee arthroplasty	2 (5.1%)	97% at 5 years	35.9%	6.2 ± 3.8
**2016**	**Sadr et al.** [2]	135 (149)	Case series (IV)	MFC 62%<br>LFC 29%<br>Trochlea 6%<br>Patella 1%<br>Others 2%	Median. 21 (12-55)	Median: 6.3 (1.9-16.8)	7.3 (2.2-25)	Grafts preserved at 4°C. Transplantation between 7 and 28 days from harvesting	Dowel, 127<br>Shell, 19<br>Dowel and Shell, 3	Press-fit or absorbable fixation	Chondral debridement (67%)<br>LBR (51%)<br>SMS (42%)<br>ORIF (10%)<br>Osteotomy (3%)<br>Hardware removal (3%)<br>OAT (3%)<br>Previous OCA (2%)<br>Extensor mechanism repair (1%)	NA	OCA revision, knee arthroplasty	12 (8%): 7 OCA revision, 3 UKA, 2 TKA	95% at 5 years<br>93% at 10 years	23%	6.1 ± 4.5
**2012**	**Lyon et al.** [40]	11 (12)	Case series (IV)	MFC 31%<br>LFC 54%<br>Patella 7.5%<br>Trochlea 7.5%	15.2 (13–20.4)	2 (1-3.4)	5.1 (1.8–8)	Transplantation between 14 and 21 days from harvesting	Dowel, 8<br>Shell, 4<br><br>Mean size 5.1 cm² (1.8-8)	Press-fit, 8<br>Metal or absorbable fixation, 4	0	0	X-ray at 2 years not showing full graft incorporation	0%	100% at last FU	0%	NA
**2009**	**Pasqual-Garrido et al.** [41]	46 (16)	Case series (IV)	NA	34 ± 9.5 (20-49)**	4.0 ± 1.8 (2.0-10.6)**	4.5 ± 2.7 (0.9-15)**	NA	Dowel<br><br>Mean size 2.4 ± 0.9 cm²**	NA	0	17: 5 HTO, 2 MAT, 10 MF**	Clinical failure and subsequent surgeries	1/16 OCA (6%): TKA	94% at last FU**	NA	14 months
**2007**	**Emmerson et al.** [42]	64 (66)	Case series (IV)	MFC 62%<br>LFC 38%	28.6 (15-54)	7.7 (2-22)	7.5	Grafts preserved at 4°C. Transplantation between 5 and 28 days from harvesting	Dowel and Shell<br><br>Mean size 7.5 cm²	Press-fit or absorbable fixation	1.7 procedures per patient - most commonly LBR	1 ACLR	OCA revision or removal, UKA or TKA	9 (13%): 6 OCA revision, 1 OCA removal, 1TKA, 1 UKA	91% at 5 years<br>76% at 10 and 15 years	10 (15%)	4.9 ± 2.4
**1994**	**Garrett et al.** [43]	17 (17)	Case series (IV)	LFC 100%	20 (16-46)	3.5 (2-9)	NA	Grafts preserved at 4°C. Transplantation within 4 days	Dowel, 10<br>Shell, 7<br><br>Size: 2 to 6 cm²	Fixation with Herbert screws	Abrasion arthroplasty, 14 (82.5%)	NA	X-ray not showing full graft incorporation or clinical failure	1 (6%): not specified different reconstructive surgery	94% at last FU	17 (100%): 1 failure + 16 hardware removal	15 months

Abbreviations: OCD: osteochondritis dissecans; OCA: osteochondral allograft transplantation; MFC: medial femoral condyle; LFC: lateral femoral condyle; HTO: high tibial osteotomy; TKA: total knee arthroplasty; UKA: unilateral knee arthroplasty; SMS: subchondral marrow stimulation; LBR: loose body removal; OAT: osteochondral autograft transplantation; ACI: autologous chondrocyte implantation. ORIF: open reduction and internal fixation; FU: follow-up; NA: not available.
Re-operation rate = failures + operations not related to the graft
**On total study cohort*
