# Supplementary material for: Fresh Osteochondral Allograft Transplantation in Osteochondritis Dissecans in the Knee Joint
Source: Life (Basel). 2021 Nov 8;11(11):1205. doi: 10.3390/life11111205 (PMC8622509; doi:10.3390/life11111205)
Supplement: Supplementary file 1 [file life-11-01205-s001.zip › Table S2.pdf]

Table S2. Clinical scores reported in the included studies of this review.

		Cotter et al. 2018	Sadr et al. 2016	Lyon et al. 2012	Pasqual-Garrido et al. 2009	Emmerson et al. 2007	Garrett et al. 1994
**18-point**		NA	Pr: 13.6 (± 2.0)<br>F: 16.8 (± 1.5)<br>P: <.001*	Pr: 12.7 (10–14)<br>F: 16.3 (10–18)	NA	Pr: 13.0 ± 1.7<br>F: 16.4 ± 2.0<br>P: <.01*	NA
**Tegner**		NA	NA	NA	Pr: 0<br>F: 6<br>P: <.001*	NA	NA
**Lysholm**		NA	NA	NA	Pr: 25<br>F: 37<br>P: .015	NA	NA
**IKDC total score**		Pr: 31<br>F: 59<br>P: <.001*	Pr: 44.2 (± 17.5)<br>F: 82.3 (± 15.8)<br>P: <.001*	NA	Pr: 31<br>F: 45<br>P: 0.15	NA	NA
**KS-F**		NA	Pr: 72.3 (± 18.6)<br>F: 95.7 (± 9.6)<br>P: <.001*	NA	NA	NA	NA
**KS-K**		NA	Pr: 81.1 (± 14.8)<br>F: 94.3 (± 8.8)<br>P: <.001*	NA	NA	NA	NA
**KOOS**	**Symptoms**	Pr: ≈52<br>F: ≈69<br>P: <.001*	NA	NA	Pr: 52<br>F: 74<br>P: .002*	NA	NA
	**Pain**	Pr: ≈50<br>F: ≈70<br>P: <.001*			Pr: 59<br>F: 67<br>P: .270		
	**ADL**	Pr: ≈61<br>F: ≈82<br>P: <.001*			Pr: 57<br>F: 67<br>P: .200		
	**Sport**	Pr: ≈23<br>F: ≈51<br>P: <.001*			Pr: 32<br>F: 46<br>P: .037*		
	**QOL**	Pr: ≈21<br>F: ≈51<br>P: <.001*			Pr: 29<br>F: 39<br>P: .062		
**WOMAC**	**Overall**	Pr: ≈38<br>F: ≈15<br>P: <.001*	NA	NA	NA	NA	NA
	**Pain**	Pr: ≈8<br>F: ≈4<br>P: <.001*					
	**Stiffness**	Pr: ≈3<br>F: ≈2<br>P: <.002*					
	**Function**	Pr: ≈25<br>F: ≈11<br>P: <.001*					
**SF-12**	**Physical**	Pr: ≈33<br>F: ≈41<br>P: <.001	NA	NA	Pr: 42<br>F: 52<br>P: .112	NA	NA
	**Mental**	Pr: ≈53<br>F: ≈53<br>P: .910			Pr: 40<br>F: 43<br>P: .370		
**VAS**		NA	NA	Pr: 5.6<br>F: 1.2	NA	Pr: 6.7 ± 2<br>F: 0.9 ± 1.3	NA
**Self-assessment function scale 10-point scale**		NA	NA	NA	NA	Pr: 3.4 ± 1.9<br>F: 8.4 ± 1.5<br>P: <.01	NA
**Satisfaction at Final FU, %** (details)		81%<br>(Es: 50%;<br>S: 31.6%)	95%<br>(Es: 78%; S: 17%;<br>Ss: 3%; Sd: 1%;<br>D: 1%)	NA	63%	92%	NA

Abbreviations: Pr, Preop. value; F, Final FU value; P, P-value; 18-point, modified Merle d'Aubigné-Postel scale; IKDC, International Knee Documentation mittee; KS-F, knee Society Score function; KS-K, Knee Society Score Knee; KOOS, Knee Injury and Osteoarthritis Outcome Score; QOL, quality of life; ADL, ities of daily living; WOMAC, Western Ontario and McMaster Universities Osteoarthritis Index; HSS, modified Hospital for Special Surgery; SF-12, 12-Item t Form Survey;VAS, Visual Activity Score; Es, Extremely satisfied; S, Satisfied; Ss, Somewhat satisfied; Sd, Somewhat dissatisfied; D, Dissatisfied; NA, not able.

**Statistically significant.*
